# Modulation of Paraoxonase 1 Activity and Asymmetric Dimethylarginine by Immunomodulatory Therapies in Multiple Sclerosis

**DOI:** 10.3390/ijms26199728

**Published:** 2025-10-06

**Authors:** Lilla Racz, Hajnalka Lorincz, Ildiko Seres, Laszlo Kardos, Gyorgy Paragh, Tunde Csepany

**Affiliations:** 1 Department of Neurology, Faculty of Medicine, University of Debrecen, 4032 Debrecen, Hungary; 2 Division of Metabolism, Department of Internal Medicine, Faculty of Medicine, University of Debrecen, 4032 Debrecen, Hungary; lorincz.hajnalka@med.unideb.hu (H.L.); seres@belklinika.com (I.S.); paragh.gyorgy@med.unideb.hu (G.P.); 3 Clinical Department of Infectious Diseases, University of Debrecen, 4032 Debrecen, Hungary; l.kardos@orvosbiostat.hu

**Keywords:** multiple sclerosis, oxidative stress, paraoxonase 1, asymmetric dimethylarginine, immunomodulatory treatment

## Abstract

**Background:** Neurodegeneration is present from the earliest stages of multiple sclerosis [MS], and oxidative stress together with mitochondrial dysfunction are key contributors to neuronal injury and disease progression. **Objective:** To investigate the role of the antioxidant enzyme paraoxonase 1 (PON1) and serum asymmetric dimethylarginine (ADMA) levels in MS across different disease subtypes and immunomodulatory treatments. **Methods:** Serum lipid levels and PON1 activity were measured and compared by disease subtype and treatment in a single-center MS cohort (N = 262; CIS = 10, RRMS = 208, PPMS = 19, SPMS = 25; 110 untreated, 152 treated) and in 91 healthy controls. ADMA levels were assessed in sera from 79 MS patients (19 untreated, 60 treated) and 31 age-matched controls. **Results:** Median serum paraoxonase (PON) and arylesterase (ARE) activity levels were 83.8 and 127.2 IU/L in MS patients versus 85.9 and 136.9 IU/L in controls, with no significant difference for PON (*p* = 0.191) but a significant reduction in ARE [*p* = 0.003]. PON activity differed significantly among disease subtypes (*p* = 0.023), with the highest levels in CIS. PON and ARE activity also varied across treatment groups (*p* = 0.038 and *p* = 0.034, respectively), with longitudinal analysis indicating a measurable effect of immunomodulatory therapy on PON activity at 10 years (*p* = 0.0136). Significant differences in enzyme activity were observed between untreated and interferon-treated patients (PON *p* = 0.0055, ARE *p* = 0.0001), with trends toward differences in ARE under natalizumab and fingolimod. ADMA levels were lower in MS patients than controls (*p* < 0.0001) and differed among treatment subgroups (natalizumab, dimethyl fumarate, glatiramer acetate, untreated RRMS). **Conclusions:** PON1 activity and ADMA levels differ between MS subgroups and under immunomodulatory treatments. Long-term therapy was associated with increased PON1 activity, while highly effective immunomodulators reduced ADMA levels. These changes may contribute to the treatment-related reduction in disease activity and attenuation of neurodegenerative processes in MS.

## 1. Introduction

Multiple sclerosis (MS) is a chronic inflammatory and neurodegenerative disorder of the central nervous system characterized by demyelination, axonal damage, and progressive disability. Globally, MS affects an estimated 2.8 million individuals, with prevalence ranging from 15 to 300 per 100,000 depending on geographic region; in Hungary, the prevalence is ~100 per 100,000, highlighting its significant public health burden [1]. Although traditionally regarded as primarily immune-mediated with a relapsing clinical phenotype, there is increasing evidence that neurodegeneration is present from the earliest stages of the disease and contributes continuously to disease progression [2,3,4]. Visible and measurable progression is the defining feature of the clinical subtype of progressing MS. Since the introduction of the McDonald criteria, the first suspected demyelinating event was distinguished clinically as an isolated syndrome (CIS) and incorporated into the traditional categories of relapsing–remitting MS (RRMS), secondary progressive MS (SPMS), primary progressive MS (PPMS) [5,6].

Oxidative stress and mitochondrial dysfunction are considered pivotal mechanisms in neuronal injury in MS [7]. Excess production of reactive oxygen species (ROS) and impaired antioxidant defense systems may accelerate axonal loss and neurodegeneration, particularly in progressive disease forms [8]. Among endogenous antioxidant systems, the serum enzyme paraoxonase 1 (PON1) plays a crucial role in protecting lipoproteins from oxidative modification and maintaining vascular and neuronal integrity [9,10]. Altered PON1 activity has been reported in various neurological and autoimmune diseases, but its role in MS and across different clinical subtypes remains insufficiently characterized [11,12,13,14].

PON1 activity is partly genetically determined. Its gene is located on the long arm q21-22.3 of chromosome 7. There are two important polymorphism sites in the structure of the human PON1 molecule, and the effect is substrate-dependent. The Gln/Arg (G/A) polymorphism at position 192, which mainly affects the activity of the PON1 enzyme [Arg is 8x more active]. Human PON1 therefore has three phenotypes: AA with high PON activity, GA with intermediate activity, and GG with low activity [15,16]. In MS, prior studies have reported inconsistent findings, with some describing increased PON activity and reduced ARE activity in patients compared to controls, while others reported decreased serum PON1 activity. Such discrepancies likely reflect differences in genetic background, disease subtype, and methodology.

In addition to oxidative stress, disturbances in nitric oxide metabolism and endothelial function have been implicated in MS pathogenesis [17]. Asymmetric dimethylarginine (ADMA), an endogenous inhibitor of nitric oxide synthase, has been proposed as a biomarker of endothelial dysfunction and neuroinflammation [18,19]. However, data regarding ADMA levels in MS patients, and particularly their modulation by immunomodulatory therapies, are limited and inconsistent [20,21].

PON1 activity is closely linked to HDL particles and associated proteins such as apoA-I, and its functional impairment may enhance lipid peroxidation. This can promote uptake of oxidized LDL by microglia and infiltrating macrophages in early MS lesions, facilitating demyelination and neurodegeneration. Concurrently, asymmetric dimethylarginine (ADMA), an endogenous inhibitor of nitric oxide synthase, promotes endothelial dysfunction and blood–brain barrier disruption. Together, these systemic alterations provide a mechanistic link from oxidative stress and vascular impairment to CNS pathology and disability progression in MS, highlighting PON1 and ADMA as relevant biomarkers of disease activity and therapeutic response [10].

Given these considerations, we hypothesized that PON1 activity and ADMA levels may be differentially altered in MS subtypes and influenced by disease-modifying therapies. The present study aimed to (i) assess serum PON1 and arylesterase (ARE) activity in patients with different MS subtypes compared with healthy controls, (ii) evaluate changes in enzyme activity under different immunomodulatory treatments, and (iii) determine serum ADMA levels in treated and untreated MS patients. By clarifying the relationship between antioxidant and nitric oxide-related pathways, this study may contribute to a better insight into how oxidative stress and nitric oxide signaling influence MS progression. No formal sample size calculation was performed, as this was an exploratory observational study, and the number of participants and tests was determined by patient availability.

## 2. Results

### 2.1. Demographic and Clinical Characteristics

The demographic and clinical features of the study population are summarized in Table 1. The cohort included 262 MS patients (CIS, RRMS, PPMS, SPMS) and 91 healthy controls. There were no significant differences in age, BMI, or lipid parameters (cholesterol, triglycerides, HDL, ApoA1, ApoB1, Lp(a)) between MS patients and controls. Subgroups differed by sample size, age, disease duration, and disability scores.

### 2.2. Serum PON1 and ARE Activity in MS

Median PON activity did not differ significantly between MS patients and controls (86.6 vs. 85.9 IU/L; *p* = 0.1909), whereas ARE activity was significantly reduced in MS (127.2 vs. 136.9 IU/L; *p* = 0.0033). Comparison among MS subtypes revealed significant differences in PON activity (*p* = 0.023), with the highest values observed in CIS patients. ARE activity also differed between MS and controls (*p* = 0.045), but not between MS subgroups (Table 1).

### 2.3. PON1 Genetic Phenotype

No AA (high activity) phenotype was detected in either MS patients or controls. The GG (low activity) phenotype predominated in both groups (controls: 89%; MS subgroups: 60–80.4%). The distribution of GA and GG phenotypes did not differ significantly between groups (Fisher’s exact test, *p* = 0.079).

### 2.4. Treatment Effects on PON1 and ARE Activity

PON1 activity was different in the groups according to the treatment type (*p* = 0.0003) and the therapeutic response (*p*=0.0458) in preliminary observations with platform therapies [22].

When grouped by disease-modifying therapy in our cohort, significant differences were observed in both PON (*p* = 0.038) and ARE activity (*p* = 0.034) (Table 2).

- Dimethyl fumarate (DMF) showed the lowest PON activity (60.41 U/mL).
- For ARE activity, natalizumab-treated patients had the lowest values (112.60 U/mL), whereas DMF-treated patients had the highest (135.65 U/mL).

**Table 2:** Characteristics of patients with different disease-modifying treatments.

	No Treatment	INF	GA	Natalizumab	Fingolimod	DMF	Immunosuppressive	*p*
Number of patients: 261	110	59	40	11	19	17	5	
Age (year) median	38	40	38.5	33	36	32	38	0.2795
EDSS median	2.5	2	2.25	4	3	1.5	3	0.2722
Paraoxonase (IU/L) median	122.28	63.30	136.63	109.00	76.78	60.41	123.04	0.0382
Arylesterase (IU/L) median	130.18	118.67	128.48	112.60	132.73	135.65	125.13	0.034

### 2.5. Serum ADMA Concentrations

**Figure 1:**
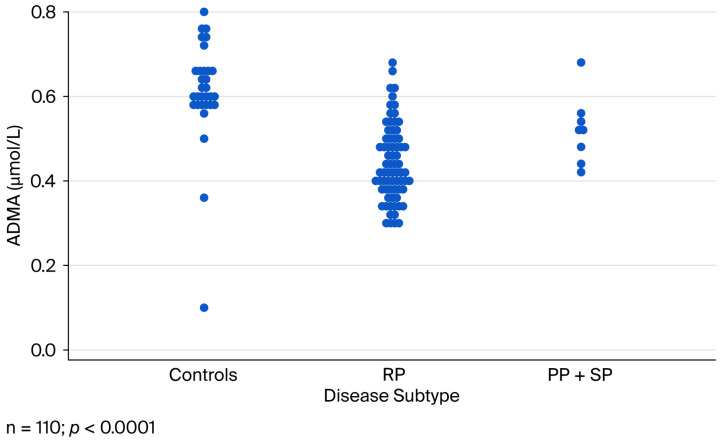
Mean ADMA values of healthy control and the MS patients. Significant differences between MS subgroups (Kruskal–Wallis test, *p* < 0.0001).

**Figure 2:**
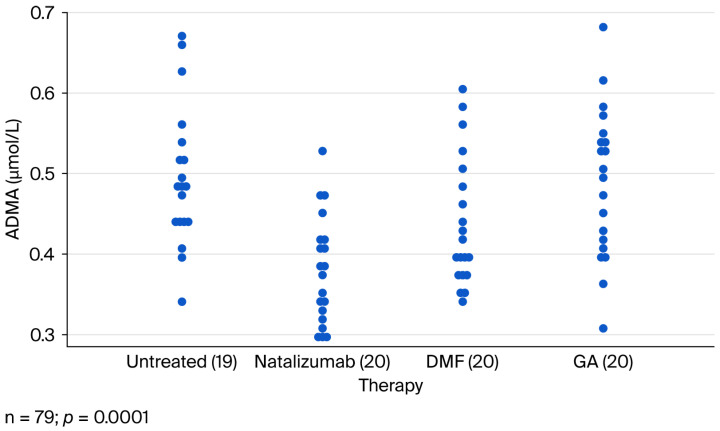
Mean ADMA values of the MS patients regarding their actual therapy (ANOVA: *p*=0.0001). Abbreviations: DMF: dimetilfumarate; GA: glatiramer acetate.

ADMA concentrations were assessed in 79 MS patients and 31 age-matched controls. Median ADMA levels were significantly lower in MS patients compared to controls (0.436 vs. 0.600 µmol/L; *p* < 0.0001). Differences were also significant between MS subgroups (Kruskal–Wallis test, *p* < 0.0001) (Figure 1). Treatment analysis revealed significant variation (ANOVA, *p* = 0.0001), with untreated patients showing the highest ADMA levels (0.495 µmol/L) and natalizumab-treated patients the lowest (0.380 µmol/L) (Figure 2).

### 2.6. Longitudinal Analysis of PON1 Activity

**Figure 3:**
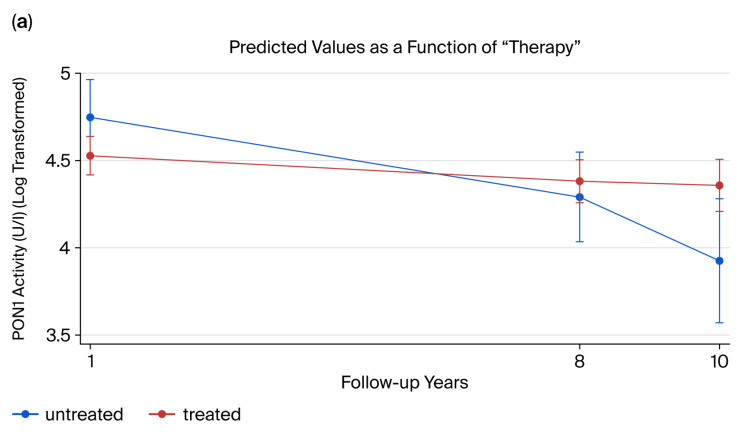

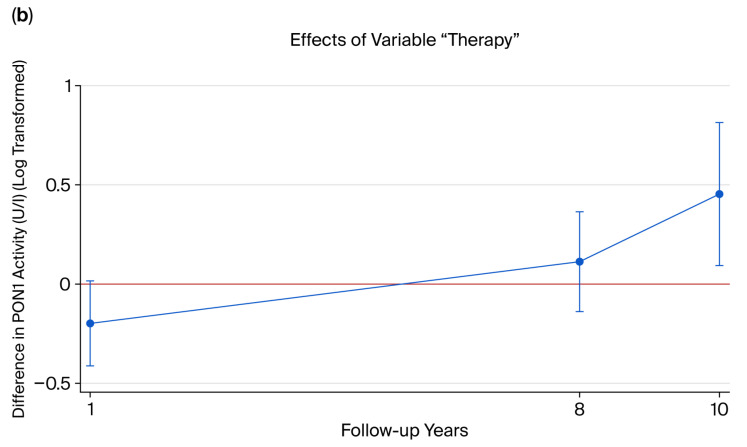
Association between PON1 activity (values were used in log-transformed form) and treatment at 8 and 10 years of follow-up. Error bars represent 95% confidence intervals. (**a**). Predicted PON1 paraoxonase activity in 8 and 10 years in untreated (blue line) and immunomodulatory-treated (red line) MS patients using longitudinal regression analysis. (**b**): Effect of immunomodulatory treatment in patients compared to those who remain untreated at 8 and 10 years of follow-up. Increased activity is observed at 10 years (*p* = 0.0136).

**Figure 4:**
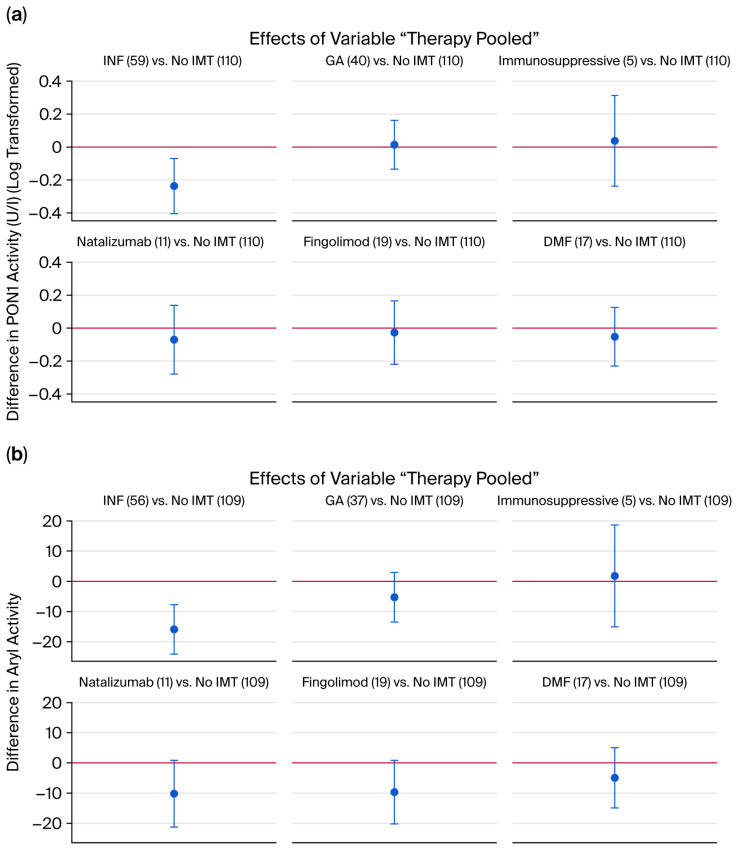
The effects of different treatments as depicted in a linear regression model; the PON1 values were used in log-transformed form. The effect of INF beta (**a**): paraoxonase [*p* = 0.0055; Cohen’s d = 0.96]; (**b**): arylesterase (*p* = 0.0001; Cohen’s d = 0.93) compared to untreated MS.

Longitudinal regression analysis adjusted for age, sex, smoking, and follow-up time demonstrated a treatment-associated increase in PON1 activity at 10 years (*p* = 0.0136; Cohen’s d = 1.89), but not at 1 year (*p* = 0.0696; Cohen’s d = 0.83) or 8 years (*p* = 0.3797 > Cohen’s d = 0.47) (Figure 3). Among individual therapies, a significant effect was observed for interferon treatment. A trend toward lower ARE activity was noted in natalizumab- and fingolimod-treated patients compared to untreated patients (*p* = 0.0707, Cohen’s d = 0.59; and *p* = 0.0723, Cohen’s d = 0.57, respectively) (Figure 4).

### 2.7. Disability and Enzyme Activity

**Figure 5:**
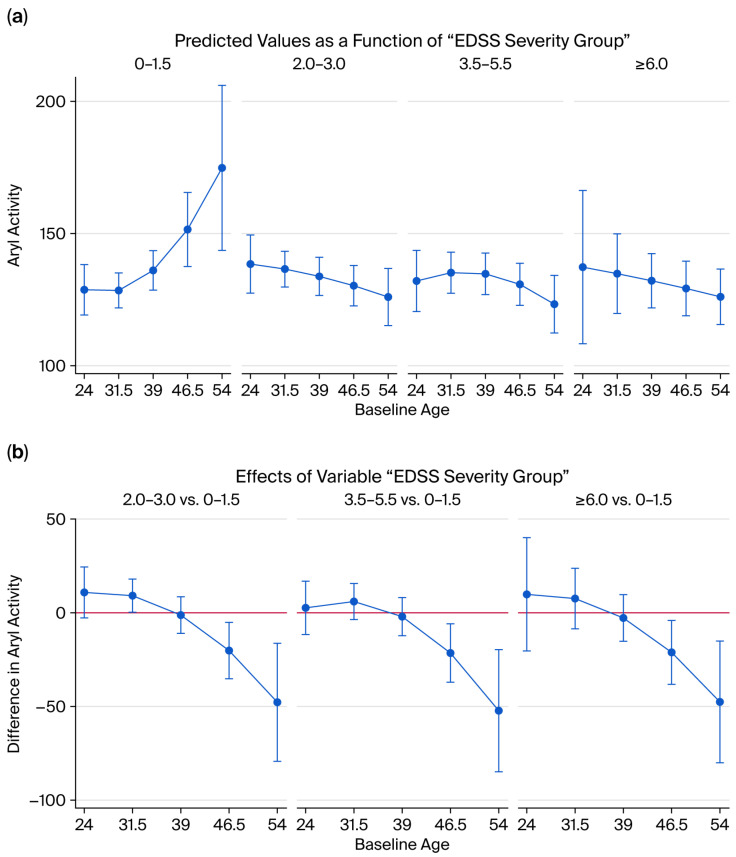
Severity of MS disease and its effects on ARE activity. (**a**): Predicted ARE activity by different EDSS categories and age at baseline. (**b**): Differences in ARE activity by different EDSS categories and age at baseline.

Analysis of Expanded Disability Status Scale (EDSS) subgroups showed no significant change in PON activity. However, ARE activity decreased with higher EDSS categories, particularly in patients >46 years of age. Older patients with mild disability (EDSS 0–1.5) had higher ARE activity, whereas in moderate-to-severe disability groups (EDSS 2.0–6.0), ARE activity was significantly lower in older patients (all *p* < 0.0154; Cohen’s d range 1.19 to 3.09) (Figure 5).

## 3. Discussion

Multiple sclerosis (MS) is a heterogeneous disorder in which disability accrual reflects a complex interplay between inflammation, oxidative stress, mitochondrial dysfunction, and neurodegeneration, further modulated by aging [7]. Despite the expansion of disease-modifying therapies, effective reversal of progression remains an unmet clinical need, and reliable biomarkers of disability accumulation are lacking.

Paraoxonase 1 (PON1) is an HDL-associated antioxidant enzyme involved in protection against lipid peroxidation and oxidative stress. Its role has been implicated in several neurodegenerative and systemic inflammatory diseases. We investigated serum PON and arylesterase (ARE) activity across MS subtypes and under different treatments. In our study, ARE activity was significantly lower in MS patients, while PON1 activity exhibited subtype-specific patterns: highest in CIS, lower in RRMS, and variable in progressive forms, with higher levels in primary progressive MS than SPMS. These findings support the hypothesis that antioxidant enzyme activity is altered early in the disease and may decline with progression.

Previous studies on PON1 in MS have yielded inconsistent results, with reports of decreased [20,23] or unchanged [24,25] activity. Variability likely reflects the heterogeneous nature of MS, small sample sizes (<200), and restriction to RRMS cohorts. Our study included the largest cohort to date across all subtypes, strengthening the reliability of subtype-related differences. We demonstrate for the first time that disease-modifying therapies lead to a sustained increase in PON1 activity over a 10-year follow-up period, with beneficial effects on the metabolic status of older patients. Although we did not observe statistically significant differences in lipid parameters (total cholesterol, LDL, HDL, triglycerides) between MS patients and healthy controls or among disease subtypes, PON1 activity remains mechanistically linked to HDL particles. Functional changes in PON1 and ARE can therefore reflect disease-relevant oxidative stress even in the absence of overt lipid abnormalities. ARE activity declined with advancing age and disability, particularly after 40–50 years, reinforcing the role of impaired antioxidant defense in later disease stages. Sex-specific effects could not be evaluated due to cohort size, though prior studies suggest higher PON1 activity in females, potentially influencing antioxidant protection. Genetic polymorphisms strongly influence PON1 activity. In our cohort, only intermediate (GA) and low (GG) phenotypes were observed, consistent with prior data from autoimmune diseases such as SLE and RA [12,14].

Treatment effects on PON1 activity have been inconsistent in the literature. While Jamroz-Wisniewska et al. reported no significant effect of mitoxantrone but improved activity with cladribine [26], our data revealed distinct activity profiles under different immunomodulatory therapies. Specifically, PON activity was reduced in patients treated with interferons, dimethyl fumarate, and fingolimod, while immunosuppressive therapy showed no change compared to untreated patients. Importantly, in our longitudinal analysis, immunomodulatory treatment was associated with a relative increase in PON1 activity after ~10 years, whereas untreated patients exhibited a marked decline. This effect was most pronounced for interferons and trended toward natalizumab and fingolimod. Whether the decline in untreated patients reflects the natural course of MS or the absence of therapy requires further study. Age-related decline in PON1 has been documented [27,28], yet our findings suggest that long-term therapy may mitigate this process in MS.

We also examined the relationship of PON1 activity to disability. Consistent with Ferretti et al. [20], low PON activity was previously linked to higher EDSS, although we did not observe such an association in our cohort. Instead, ARE activity declined with advancing age and disability, particularly after 40–50 years, reinforcing the role of oxidative stress in later disease stages.

Beyond PON1, we analyzed asymmetric dimethylarginine (ADMA), an endogenous inhibitor of nitric oxide synthase that promotes endothelial dysfunction and blood–brain barrier disruption. Elevated ADMA has been linked to relapse, disability, and longer disease duration [21,29,30,31]. In contrast, we observed lower ADMA levels in MS compared to controls, with significant differences across treatment subgroups. Notably, natalizumab was associated with the most pronounced reduction in ADMA, consistent with its high efficacy in reducing inflammation and protecting the CNS [32,33,34]. These findings suggest that ADMA may serve as a mechanistic marker of endothelial dysfunction that responds to potent immunomodulatory therapy.

Taken together, our results indicate that PON1 activity and ADMA levels differ across MS subtypes and treatment groups. Long-term immunomodulatory therapy was associated with preserved or increased PON1 activity, while highly effective therapy, particularly natalizumab, was linked to reduced ADMA levels. These biomarker changes likely reflect attenuation of oxidative stress and endothelial dysfunction, processes central to MS pathophysiology and neurodegeneration. Further studies are warranted to validate PON1 and ADMA as biomarkers of disease progression and treatment response in larger, multicenter cohorts.

## 4. Materials and Methods

### 4.1. Study Population

The MS Center at the Department of Neurology, University of Debrecen, was established in 1992. Patients have been diagnosed with multiple sclerosis (MS) according to the McDonald criteria since 2001 [5]. Clinical documentation included disease onset and diagnosis dates, MS course, relapse activity, Expanded Disability Status Scale (EDSS) scores [35], cerebrospinal fluid analysis, MRI, visual evoked potentials, laboratory tests, and treatment history.

Specific immunotherapies became available from 1996, followed by natalizumab in 2010, and subsequent disease-modifying therapies were introduced into routine care with a 3–4-year delay after European Medicines Agency approval. Treated patients underwent regular follow-up every three months and annual MRI. Progression was defined as a confirmed EDSS increase. Patients were classified as treatment-responsive or non-responsive, and therapy was changed when possible [36].

No formal sample size calculation was performed prior to the study, as this was an exploratory observational analysis. The number of participants was determined by the availability of eligible patients during the recruitment period and the extent of laboratory testing. While this approach may limit statistical power for some subgroup comparisons, the total cohort represents one of the largest to date in which paraoxonase 1 activity and asymmetric dimethylarginine were analyzed across all multiple sclerosis subtypes.

Study Design: This was a cross-sectional observational study conducted at our clinic. Serum paraoxonase 1 (PON1), arylesterase (ARE) activity, and asymmetric dimethylarginine (ADMA) levels were measured in patients with multiple sclerosis (MS) and compared with healthy controls. Variables of interest included disease subtype (CIS, RRMS, SPMS, PMS), treatment status (untreated vs. immunomodulatory therapy), and clinical disability assessed by the Expanded Disability Status Scale (EDSS).

Constraints and Funding: The number of tests performed was dependent on the availability of research. As a result, not all patients underwent all assays, which limited subgroup sizes for certain analyses. This represents a constraint of the present study but does not affect the overall validity of the comparisons made.

Participants: MS patients were consecutively recruited from our outpatient cohort. The inclusion criteria were an age of 18–70 years, a diagnosis of MS according to the McDonald criteria, and the availability of clinical data and serum samples. The exclusion criteria were an acute infection within the previous 4 weeks, other autoimmune or systemic inflammatory diseases, diabetes mellitus, chronic liver or kidney disease, or the use of lipid-lowering medication. Healthy controls were age- and sex-matched volunteers without neurological or systemic diseases and not on chronic medication.

Plasma samples were collected from 215 patients in 2012, with follow-up collections in 2018 (*n* = 26) and 2019 (*n* = 157, including 84 previously studied and 73 new patients). At each sampling, demographic and clinical data were recorded, including gender, age, MS type, disease duration, BMI, EDSS, current treatment, smoking status, and serum lipid levels. Patients were stratified by EDSS: group A (0–1.5), group B (2.0–3.0), group C (3.5–5.5), and group D (≥6.0). Treatment groups included interferon (IFN), glatiramer acetate (GA), natalizumab, fingolimod, siponimod, dimethyl fumarate (DMF), and immunosuppressive therapies (teriflunomide, mitoxantrone, azathioprine, daclizumab, alemtuzumab, ocrelizumab, cladribine). Untreated patients consisted of relapsing MS patients with minimal symptoms or patients with progressive MS (PP, RP, SP).

All participants provided written informed consent. The study was approved by the Regional Ethical Committee of the University of Debrecen (DE RKEB/IKEB: reference number 3941-2013, accepted: 17 June 2013; reference number 5224-2019, accepted: 19 April 2019)) and conducted in accordance with the Declaration of Helsinki.

### 4.2. Blood Collection and Lipid Measurements

Fasting venous blood samples (10 mL) were collected between 07:30 and 08:00, centrifuged at 1500 g for 10 min, and hemolysis-free serum was stored. Lipid measurements were performed on fresh serum, while samples for PON1 analysis were stored at −70 °C.

Serum cholesterol and triglycerides were determined from fresh sera using enzymatic colorimetric assays (GPO-PAP, Modular P-800 Analyzer, Roche/Hitachi). HDL-C was measured with a homogeneous enzymatic colorimetric assay (Roche HDL plus, 3rd generation). LDL-C was calculated using the Friedewald equation [26]. Apolipoproteins A1 and B were measured by immunoturbidimetric assays (Tina-Quant ApoA1 and ApoB, Roche).

### 4.3. Measurement of Serum Paraoxonase (PON1) and Arylesterase (ARE) Activity

PON activity was measured using paraoxon as a substrate, and the formation of 4-nitrophenol was monitored spectrophotometrically at 412 nm (25 °C) [11]. Activity was calculated using a molar extinction coefficient of 17,100 mol^−1^ cm^−1^, expressed as nmol of 4-nitrophenol produced per minute (international unit per liter [IU/L]) [11].

ARE activity was determined spectrophotometrically using phenyl acetate as substrate [11]. Absorbance was recorded at 270 nm, and enzyme activity was expressed in U/mL (µmol phenyl acetate hydrolyzed per minute), using a molar extinction coefficient of 1310 mol^−1^ cm^−1^ (international unit per liter [IU/L]) [37].

### 4.4. PON1 Phenotyping

PON1 phenotypic distribution was determined using the double substrate method [38]. The ratio of salt-stimulated PON activity (in the presence of 1 M NaCl) to ARE activity classified subjects into three phenotypes: GG (low activity, ratio < 3.0), GA (intermediate, ratio 3.0–7.0), and AA (high activity, ratio > 7.0) [15].

### 4.5. Measurement of ADMA

Serum asymmetric dimethylarginine (ADMA) concentrations were measured using a commercially available ELISA kit (ADMA-ELISA, DLD Diagnostika GmbH, Hamburg, Germany), according to the manufacturer’s protocol [39].

### 4.6. Statistical Analysis

Statistical analysis was performed using Stata v12. A *p*-value < 0.05 was considered statistically significant. Group comparisons were carried out using Fisher’s exact test for categorical variables and one-way ANOVA or Kruskal–Wallis test for continuous variables, depending on distribution. Spine and dot plots were used for visualization. Relationships between continuous variables and enzyme activity were assessed using scatter plots with LOWESS smoothing.

To account for repeated measures, multilevel mixed-effects linear regression models were applied to evaluate the effect of explanatory variables on PON and ARE activity. Models were adjusted for follow-up time, sex, age, smoking, ApoB, EDSS category, and MS subtype, if inclusion improved model fit. Interaction terms and nonlinear effects were explored when relevant. Effect sizes were expressed as Cohen’s d, for which the pooled standard deviation was estimated by the mixed-effects model.

## 5. Conclusions

Our study demonstrates the following:

1. PON1 and ARE activity show subtype-specific alterations in multiple sclerosis, with the lowest values observed in progressive disease. 2. Long-term immunomodulatory therapy preserves or increases PON1 activity, while untreated patients exhibit a decline over time. 3. ADMA levels are reduced in treated patients, particularly under natalizumab, suggesting therapy-linked improvement in endothelial function. 4. PON1 activity and ADMA can be assessed from peripheral blood, highlighting their potential as practical biomarkers for monitoring disease progression and treatment response.

Future research should aim to validate PON1 activity and asymmetric dimethylarginine as biomarkers of oxidative stress and endothelial dysfunction in multiple sclerosis using larger, multicenter cohorts. Longitudinal studies are needed to determine whether therapy-induced changes in these biomarkers predict long-term disability outcomes. Stratification by sex, age, and genetic polymorphisms will be essential to clarify inter-individual variability. Finally, therapeutic approaches that enhance PON1 activity or reduce ADMA levels may represent novel adjunctive strategies to attenuate oxidative and vascular injury in multiple sclerosis, and merit systematic investigation.

## Figures and Tables

**Table 1:** Patient characteristics as well as PON and ARE activity in subtypes of MS patients by Wilcoxon rank-sum test.

	Disease Subtype					
	CIS	RR	PP+RP	SP	Total	*p*
Total [N]	10	208	19	25	262	
Age (year) mean ± SD	36.2 ± 9.3	36.7 ± 9.9	46 ± 9.6	50.96 ± 9.0	38.6 ± 10.7	<0.0001
Duration of disease (year) median	0	4.5	4	14	5	<0.0001
EDSS (median)	2.0	2.0	4.5	7.0	2.0	<0.0001
HDL-C (mM/L) median	1.37	1.4	1.4	1.3	1.4	0.4015
LDL-C (mM/L)	2.59	2.88	2.9	2.92	2.9	0.9190
ApoA1 (g/L)	1.47	1.51	1.5	1.33	1.48	0.0775
ApoB (g/L) median	0.78	0.8	0.86	0.83	0.81	0.4057
Lp(a) (mg/L) median	228	110	156	130	116	0.3534
BMI (kg/m^2^) median	23	24	25	24	24	0.669
Paraoxonase (IU/L) median	196.3	74.9	176.5	99.3	86.6	0.0231
Arylesterase (IU/L) median	124.6	126.8	122.4	131.5	127.2	0.7728

## Data Availability

The data presented in the study are available on request from the corresponding author.

## Abbreviations

The following abbreviations are used in this manuscript:

MS	Multiple sclerosis
PON1	Paraoxonase 1
ADMA	Asymmetric dimethylarginine
CIS	Clinically isolated syndrome
RRMS	Relapsing-remitting multiple sclerosis
PPMS	Primary-progressive multiple sclerosis
SPMS	Secondary-progressive multiple sclerosis
RPMS	Relapsing-progressive multiple sclerosis
ARE	Arylesterase
ROS	Reactive oxygen species
EDSS	Expanded Disability Status Scale
BMI	Body mass index
IFN	Interferon
GA	Glatiramer acetate
DMF	Dimethyl fumarate
